# Supplementary material for: Vaccination: Adherence and Hesitancy among Pregnant Women for COVID-19, Pertussis, and Influenza Vaccines
Source: Vaccines (Basel). 2024 Apr 17;12(4):427. doi: 10.3390/vaccines12040427 (PMC11054588; doi:10.3390/vaccines12040427)
Supplement: Supplementary file 1 [file vaccines-12-00427-s001.zip › vaccines-2903483-supplementary.pdf]

Supplementary Materials

QUESTIONNAIRE ON VACCINATION DURING PREGNANCY

Biographical data

First name; Last name; Date of birth; Age; Nationality; Level of education Occupation

Obstetrical and clinical history:

Parity (how many children do you have at home?)

Pregnancy followed by

- High-risk pregnancy outpatient clinic
- General pregnancy outpatient clinic
- Private gynaecologist
- Midwife
- Other (please specify) ___________________

Are you taking any medications? Yes/No

If yes, which ones?

Do you have any allergies?

If yes, which ones?

1. **For which of these vaccinations have you received/requested information during your current pregnancy?**

- SARS-CoV-2
- Pertussis
- Influenza
- None
- Other (please specify) ___________

2. Have you been vaccinated during your current pregnancy? Yes/No

3. Which vaccination did you receive during your current pregnancy?

- SARS-CoV-2
- Pertussis
- Influence
- Other (please specify) ___________________
- None

4. Do you plan to get vaccinated during your current pregnancy? Yes/No

5. If yes, which vaccine do you plan to receive?

- SARS-CoV-2
- Pertussis
- Influence
- Other (please specify) ___________________
- None
- All vaccines listed

6. Have you been vaccinated in the past? Yes/No

7. If yes, were you pregnant when you were vaccinated? Yes/No

8. Did you have any side effects as a result of the vaccination? Yes/No

9. If yes, what were they?

- Fever
- Cough
- Muscle pain
- Vomiting
- Other (please specify) ________________

10. If you were NOT vaccinated, please indicate reasons among those listed (multiple answers possible)

Logistical problems

- I did not have time to go to the vaccination centre
- I did not get enough information about the where, when and how to get vaccinated
- There was no vaccine available
- I did not have the opportunity to go to/be accompanied to the vaccination centre

Personal history

- I was sick after a previous vaccination
- I was sick after a previous vaccination received during pregnancy
- I have a chronic illness that is a contraindication to vaccination
- I have a chronic illness because of which I am afraid to be vaccinated
- A family member/friend of mine was sick after a vaccination
- I am taking medication that constitutes contraindication to vaccination

Influence by other people

- No health professional has given me the information I need to get vaccinated
- My partner/husband has advised me not to get vaccinated during pregnancy
- Friends/relatives have advised me not to get vaccinated during pregnancy
- I have read on the internet/newspaper articles that it is not recommended to get vaccinated during pregnancy
- It was reported on TV that the vaccine is dangerous and that it is not recommended to get vaccinated during pregnancy

Personal belief

- I am afraid that the vaccine could be dangerous for my health
- I am afraid that the vaccine could be dangerous for my baby's health
- I do not need the vaccine because I do not have contact with people who are at risk of getting ill

11. Perception of risk - Influenza

- Is it less likely to get influenza during pregnancy? Yes/No
- Can influenza during pregnancy be dangerous to the mother and foetus? Yes/No
- Are women who get influenza during pregnancy at higher risk of being hospitalised? Yes/No
- Is the vaccine dangerous to the foetus? Yes/No
- Am I afraid of the side effects of the vaccine during pregnancy? Yes/No
- Am I afraid of the effects of influenza on me if I contract it during pregnancy? Yes/No
- Am I afraid of the effects of influenza on the foetus if I contract it during pregnancy? Yes/No
- Is the vaccine unable to protect me from influenza? Yes/No
- Will the vaccine protect me from contracting influenza? Yes/No

12. Risk perception - Pertussis (answer yes/no to the following questions)

- Is it dangerous to get pertussis during pregnancy? Yes/No

- Can pertussis be very dangerous for the newborn? Yes/No
- Can the infant die in the first few months after birth if I have not been vaccinated against pertussis? Yes/No
- Can the pertussis vaccine be dangerous for me? Yes/No
- Can the pertussis vaccine be dangerous to the foetus? Yes/No
- Will the pertussis vaccine protect my child in the first few months after birth? Yes/No

13. Risk perception - Rubella

- Did you get vaccinated against rubella before becoming pregnant? Yes/No
- Were you informed about the importance of this vaccination? Yes/No

14. Knowledge about vaccines knowledge (identify true/false statements among the following):

- Not enough is known about the vaccine
- Vaccines have not been studied enough
- It is more dangerous to get vaccinated than to contract the disease
- If everyone gets vaccinated, I do not need to get vaccinated
- I would like to get more information about vaccination from my doctor/gynaecologist

15. In what ways would you like to receive information about vaccination during pregnancy?

- Leaflet
- From my gynaecologist
- From the general doctor
- Other (please specify) ________________________
